# Prognostic Significance of Preoperative Sarcopenia in Patients With Gastric Cancer Liver Metastases Receiving Hepatectomy

**DOI:** 10.3389/fnut.2022.878791

**Published:** 2022-05-10

**Authors:** Jianping Xiong, Yunzi Wu, Haitao Hu, Wenzhe Kang, Yang Li, Peng Jin, Xinxin Shao, Weikun Li, Yantao Tian

**Affiliations:** Department of Pancreatic and Gastric Surgery, National Cancer Center/ National Clinical Research Center for Cancer/Cancer Hospital, Chinese Academy of Medical Sciences and Peking Union Medical College, Beijing, China

**Keywords:** gastric cancer, liver metastases, hepatectomy, sarcopenia, prognostic factors

## Abstract

**Background:**

The present work focused on assessing the role of computed tomography (CT)-determined sarcopenia in the prognosis of patients with gastric cancer liver metastases (GCLM) receiving hepatectomy.

**Methods:**

We analyzed data collected from GCLM cases that underwent hepatectomy between March 2011 and July 2017. The third lumbar vertebra (L3) level skeletal muscle index (SMI) was analyzed by abdominal CT to determine the sarcopenia before surgery. The thresholds for CT-based sarcopenia of sex-specific L3 SMI were ≤ 34.9 cm^2^/m^2^ and ≤ 40.8 cm^2^/m^2^ for female and male, separately We determined overall survival (OS) and recurrence-free survival (RFS)by univariate and multivariate analyses.

**Results:**

The cohort enrolled altogether 114 patients with GCLM receiving hepatectomy (average age: 62.6 years, male: 79.8%), and 58 (50.8%) patients had sarcopenia. The mean SMI was 34.2 in patients with sarcopenia compared to 42.7 in patients without sarcopenia (*p* < 0.001). The 1-, 3-, and 5-year OS rates in patients with GCLM after hepatectomy were 78.1, 43.7, and 34.3%, respectively. The 1-, 3-, and 5-year RFS rates in patients were 49.8, 33.6, and 29.3%, respectively. Sarcopenia was related to an advanced age (≥65.0 years) (*p* = 0.009), reduced BMI (<18.5 kg/m^2^) (*p* < 0.001) and number of liver metastases (>1) (*p* = 0.025). Sarcopenia had a significant associated with the patterns of recurrence (*p* < 0.001). In addition, patients with sarcopenia had a significant difference in number of liver metastases in comparison with those without sarcopenia (*p* = 0.025). We discovered from multivariate analysis that sarcopenia independently predicted RFS [hazard ratio (*HR*) = 1.76; 95% confidence interval (*CI*)= 1.18–2.35, *p* = 0.007]. Nevertheless, sarcopenia was not the prognostic factors that independently predicted OS (*HR* = 1.62; 95% *CI* = 0.57–2.73; *p* = 0.330).

**Conclusions:**

In conclusion, we showed that CT-determined sarcopenia was the facile and effective prognostic factor for RFS inpatients with GCLM after hepatectomy. Patients with sarcopenia are associated with an increased tumor recurrence risk, and thereby customized treatment should be applied.

## Introduction

Gastric cancer (GC) ranks the 5th place among cancers in terms of its morbidity, and there are 1,033,701 patients being diagnosed annually. Also, GC is the 3rd most common reason for cancer-associated mortality, which causes about 782,685 deaths every year (1). Although great achievements have been made in diagnosing and treating GC, distant metastases are related to reduced survival. The liver is the common organ of distant metastases from gastric cancer and the incidence of GC liver metastases (GCLM) is 9.9–18.7% (2, 3). The median survival time of patients with GCLM is about 7–12 months (4). Even with first-line chemotherapy, it is difficult for patients with GCLM to achieve long-term survival (4). Considering the dismal overall survival results receiving palliative chemotherapy alone, hepatectomy has been investigated as a suitable measure to improve outcome. Many studies have described the advantage of liver resection for GCLM over the past 2 decades (5–7). Recently, several large-scale studies have reported that 5-year overall survival rates were 31.1–39.5% of liver resection for GCLM (8, 9). Nevertheless, due to the extremely high recurrence rate after hepatectomy, it is difficult to determine the indication and timing of hepatectomy for GCLM. Therefore, assessment approaches for hepatectomy for GCLM are necessary.

Sarcopenia, which is a kind of age-associated losses of muscle strength, mass, as well as function, has become a serious medical issue in aging societies (10). Sarcopenia is significantly related to non-alcoholic fatty liver disease, liver cirrhosis or cardiovascular events (11–13). It is recognized that more and more attention has been paid to the impact of sarcopenia on the prognosis of patients with cancer, since low muscularity represents an important predicting factor for dismal survival of different tumors (14, 15). Sarcopenia is frequently seen among patients with GC, with a prevalence of over 6.8–57.7% in the patients with GC (16). And it is markedly related to the dismal long-run prognostic outcome in GC cases undergoing surgical resection (17). Nevertheless, the importance of sarcopenia in predicting the prognostic outcome of patients with GCLM after hepatectomy has not been reported.

In this study, the aim of the present work was to assess the significance of sarcopenia in patients with GCLM after hepatectomy, and exploring the relation of sarcopenia with additional clinicopathological characteristics. We also examined whether sarcopenia could become one of the assessment approach for hepatectomy.

## Methods

The Institutional Review Board of National Cancer Center/Cancer Hospital, Chinese Academy of Medical Sciences and Peking Union Medical College approved our study (NCC2020C-220). This work was performed following the Declaration of Helsinki and the Transparent Reporting of a Multivariable Prediction Model for Individual Prognosis or Diagnosis (TRIPOD) reporting guideline.

### Study Design and Population

**Figure 1:**
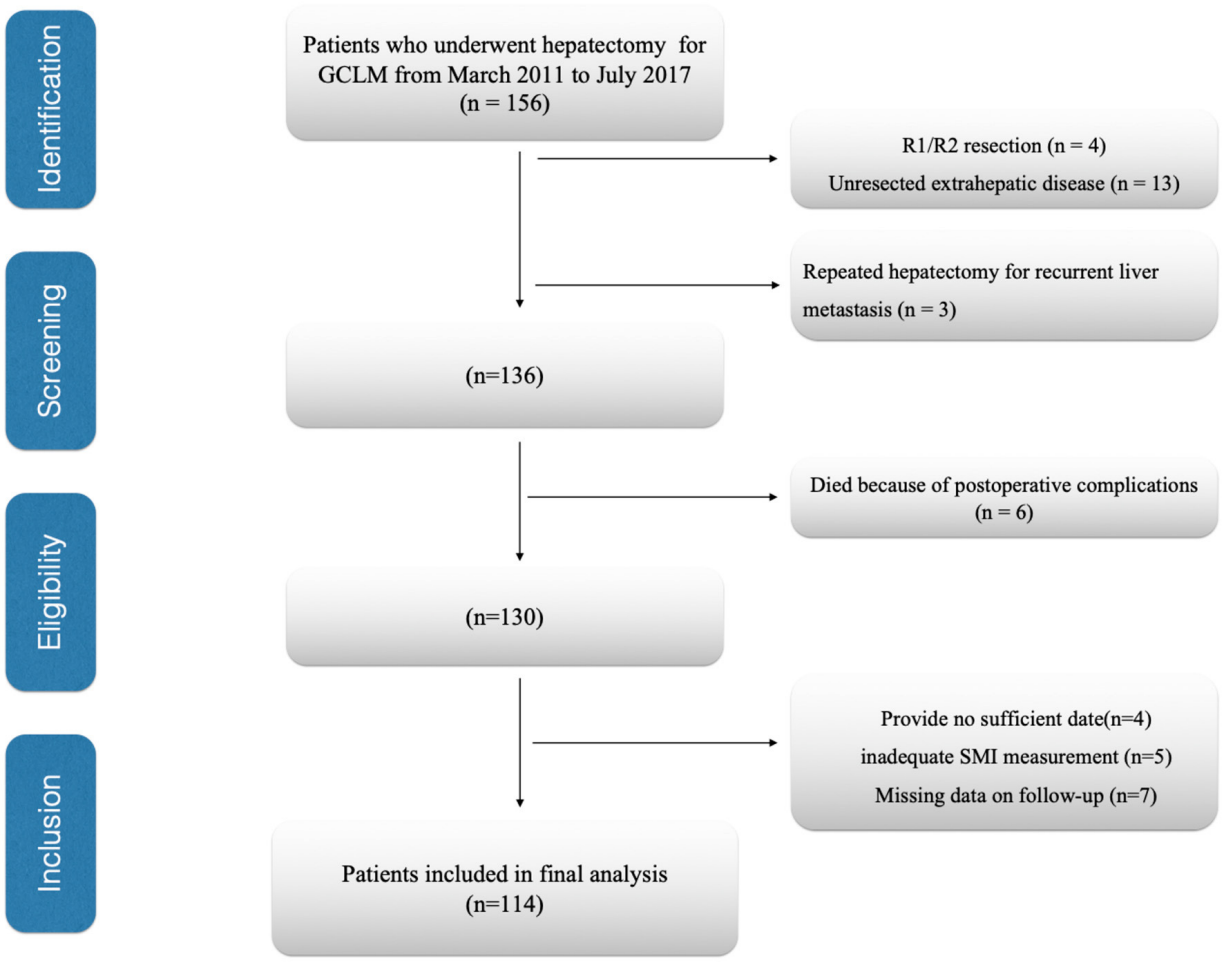
Flow diagram of patients. GCLM, gastric cancer liver Metastasis; SMI, skeletal muscle index.

The present work evaluated all cases receiving liver resection for GCLM at the National Cancer Center/Cancer Hospital, Chinese Academy of Medical Sciences and Peking Union Medical College from March 2011 to July 2017. Patients conforming to the criteria below were excluded: (1) unresected extrahepatic disease, (2) repeated hepatectomy for recurrent liver metastases, (3) those receiving R1/R2 resection, (4) those with insufficient/inexact medical records, (5) those with inadequate skeletal muscle index (SMI) measurement, (6)died because of postoperative complications, and (7) patients who had insufficient follow-up data. Finally, we enrolled 114 cases into the cohort (Figure 1). Additionally, we also analyzed patients' laboratory, demographic and histopathological data and collected related data based on patient records in this institute and relevant databases. The collected data included age, sex, body mass index (BMI, kg/m^2^), CEA levels, serum albumin, American society of anesthesiologists (ASA) score, location of gastric cancer, Lauren classification, tumor differentiation, timing of liver metastases (metachronous or synchronous), number of liver metastases, maximum diameter of the liver metastases, type of hepatectomy, neoadjuvant chemotherapy, adjuvant chemotherapy, and survival. Major hepatectomy was considered as the number of resection liver segments ≥3, where as minor hepatectomy was considered as the number of resection liver segments <3 (18). Post-operative follows-up were conducted at 3-month intervals in initial 2 years postoperatively, and every 6 months since then. We conducted the final follow-up visit in April 2021. In follow-up visits, we examined tumor markers (CA19-9, CEA, AFP), annual endoscopy, abdomino pelvic computed tomography, and chest X-ray. The present work defined overall survival (OS) as duration between surgery date and final follow-up or all-cause mortality, which served as a primary endpoint, while recurrence-free survival (RFS) as duration between surgery date and disease recurrence or mortality, and it served as a secondary endpoint. We recorded all-cause mortality as an event.

### Definition of Sarcopenia

According to latest Asian Working Group for Sarcopenia (AWGS) guideline, sarcopenia was considered as low muscle quality plus low grip strength or slow gait speed (19). Because our study design was retrospective in nature, it was not possible to collect information on muscle function (muscle strength or physical performance). Therefore, we preoccupied with muscle quality assessment to identify patients with sarcopenia. Computed tomography (CT) is used as a means to accurately assess muscle mass. CT images at the beginning of the treatment (before neoadjuvant chemotherapy) were retrieved for analysis. By adopting a public semi-automatic software (BMI measurement approach, version 1.0; https://sourceforge.net/projects/ muscle-fat-area-measurement/), we determined the cross-section areas (CSAs) of paraspinal muscles, psoas muscles, as well as rectus, oblique and transverse abdominal muscles at the third lumbar vertebra (L3) level with the threshold being−29–150 Hounsfield units (HU) (20, 21). The radiologist who had 5-year experience of abdominal imaging and was blinded to subject information was invited for analyses by the de-identified Digital Imaging and Communications in Medicine files. We later normalized the L3 skeletal muscle index (SMI) to patient stature below: lumbar total muscle CSA (cm^2^)/height (m^2^). In addition, the thresholds for CT-based sarcopenia of Sex-specific L3 SMI were ≤ 34.9 cm^2^/m^2^ and ≤ 40.8 cm^2^/m^2^ for female and male, separately, which was created by the Zhuang and colleagues for the Chinese population (22).

### Statistical Methods

Chi-square tests and *t* tests were used to analyze categorical and continuous data, respectively. Thereafter, we plotted the Kaplan–Meier (K-M) survival curve and examined heterogeneities in curves by log-rank test. Upon univariate analysis, we included significant variables for multivariate analysis by using Cox regression model. *p* < 0.05 (two-sided) stood for statistical significance. Statistical analyses were completed using Rver. 4.0.2 (R Foundation for Statistical Computing, Vienna, Austria), SPSS 18.0 (SPSS Inc., Chicago, IL, USA) and GraphPad Prism 7 software (GraphPad Software, CA, USA).

## Results

### Patient Features

The cohort enrolled altogether114 patients with GCLM receiving hepatectomy, including 91 (79.9%), men along with23 (20.1%) women. Their mean age when the liver resection was performed was 62.6 years [interquartile range: 57.5–70.2 years]. According to our thresholds, 58 (50.9%) cases had sarcopenia. Concerning tumor location of the primary GC, 32 patients (28.1%) had an upper lesion, and 82 patients (71.9%) had a middle or lower lesion. Concerning Lauren classification of the primary GC, 55 patients (48.3%) had anintestinal-type, 38 patients (33.3%) had a diffused-type, and 21 patients (18.4%) had mixed-type. Concerning liver metastases, 45 patients (39.5%) had synchronous metastases, and 69 patients (60.5%) had metachronous metastases. About 71 patients (62.3%) had solitary liver metastases and 43 patients (39.4%) had multiple liver metastases. About 64 patients (56.2%) received neoadjuvant chemotherapy and 75 patients (65.7%) received adjuvant chemotherapy. The 1-, 3-, and 5-year OS rates in patients with GCLM receiving hepatectomy were 78.1, 43.7, and 34.3%, respectively. The 1-, 3-, and 5-year RFS rates in patients with GCLM receiving hepatectomy were 49.8, 33.6, and 29.3%, respectively.

### Associations Between CT-Determined Sarcopenia and Clinicopathological Features

We classified patients into 2 groups based on whether they had sarcopenia. The mean SMI was 34.2 in patients with sarcopenia compared to 42.7 in patients without sarcopenia (*p* < 0.001). Table 1 displays the associations between sarcopenia and clinicopathological features in the cohort. Sarcopenia was markedly related to an advanced age (≥65.0 years) (*p* = 0.009) and reduced BMI (<18.5 kg/m^2^) (*p* < 0.001). In addition, patients with sarcopenia had a significant difference in number of liver metastases in comparison with those without sarcopenia (*p* = 0.025). The incidence rate of recurrence in the remnant liver was 53.2 in patients with sarcopenia compared to 75.6 in patients without sarcopenia (Table 2). Sarcopenia had a significant associated with the patterns of recurrence (*p* < 0.001) (Table 2).

**Table 1:** Association of Sarcopeniaand clinicopathological characteristics in patients with GCLM after hepatectomy.

**Clinicopathological features**	**Allcases (*n* = 114)**	**Sarcopenia (*n* = 58)**	**Non-Sarcopenia (*n* = 56)**	***p*** **-value**
Age				
≥ 65.0 < 65.0	62 (54.4) 52 (45.6)	37 (63.8) 21 (36.2)	25 (44.6) 31 (54.4)	0.009
Gender				
Male Female	91 (79.8) 23 (20.2)	46 (79.3) 12 (20.7)	45 (80.4) 11 (19.6)	0.603
BMI (kg/m^2^)				
< 18.5 ≥ 18.5	20 (17.5) 94 (82.5)	15 (25.8) 45 (74.2)	5 (8.9) 51 (91.1)	<0.001
L3 SMI (cm^2^/m^2^), median	38.5	34.2	42.7	< 0.001
Serum albumin (g/dL)				
≥ 3.5 < 3.5	86 (75.4) 28 (24.6)	42 (72.4) 16 (27.6)	44 (78.6) 12 (21.4)	0.102
CEA(ng/mL)				
≥ 5.0 < 5.0	60 (52.6) 54 (47.4)	33 (56.9) 25 (43.1)	27 (48.2) 29 (51.8)	0.098
ASA score				
1 2 3	11 (9.6) 90 (79.0) 13 (11.4)	5 (8.6) 46 (79.4) 7 (12.1)	6 (10.7) 44 (78.6) 6 (10.7)	0.513
Tumor locationof GC				
Upper Middle/Lower	32 (28.1) 82 (71.9)	17 (29.3) 41 (70.7)	15 (26.8) 41 (73.2)	0.466
Lauren Classification				
Intestinal-type Diffused-type Mixed	55 (48.3) 38 (33.3) 21 (18.4)	27 (46.7) 20 (34.4) 11 (18.9)	28 (50.0) 18 (32.1) 10 (17.9)	0.239
Tumor differentiation of GC				
G1 G2 G3	26 (22.8) 51 (44.7) 37 (32.5)	12 (20.3) 26 (44.8) 20 (34.9)	14 (25.0) 25 (44.7) 17 (30.3)	0.109
Timing of liver metastases				
Metachronous Synchronous	69 (60.5) 45 (39.5)	36 (62.1) 22 (37.9)	33 (58.9) 23 (41.1)	0.213
Number of liver metastases				
≤ 1 >1	71 (62.3) 43 (37.7)	33 (56.9) 25 (43.1)	38 (67.8) 18 (32.2)	0.025
Maximum diameter of the liver metastasis				
<3 ≥ 3	69 (60.6) 45 (39.4)	34 (58.6) 24 (41.4)	35 (62.5) 21 (37.5)	0.116
Type of hepatectomy				
Minor Major	88 (77.2) 26 (22.8)	44 (75.8) 14 (24.2)	44 (78.6) 12 (21.4)	0.720
Neoadjuvant chemotherapy				
No Yes	50 (43.8) 64 (56.2)	27 (46.5) 31 (53.5)	23 (41.1) 33 (58.9)	0.331
Adjuvant chemotherapy				
No Yes (Systemic chemotherapy/HAIC/ Systemic chemotherapy+HAIC)	39 (34.3) 75 (65.7)	18 (31.1) 40 (68.9)	21 (37.9) 35 (62.5)	0.175

*GCLM, Gastric cancer liver metastasis; BMI, Body mass index; ASA, American society of anesthesiologists; GC, Gastric cancer; HAIC, Hepatic artery infusion chemotherapy*.

**Table 2:** Impact of sarcopenia on the recurrence after hepatectomy for GCLM.

	**Allcases (*n* = 84)**	**Sarcopenia (*n* = 47)**	**Non-Sarcopenia (*n* = 37)**	**P-value**
Patterns of recurrence Remnant liver Extrahepatic sites (Lung/Lymph node/Peritoneal dissemination/Local (primary site)/Bone/brain)	53 (63.1) 31 (36.9)	25 (53.2) 22 (46.8)	28 (75.6) 9 (24.4)	*p*< 0.001

*GCLM, Gastric cancer liver metastasis*.

### Prognostic Factors for OS and RFS After Hepatectomy

**Figure 2:**
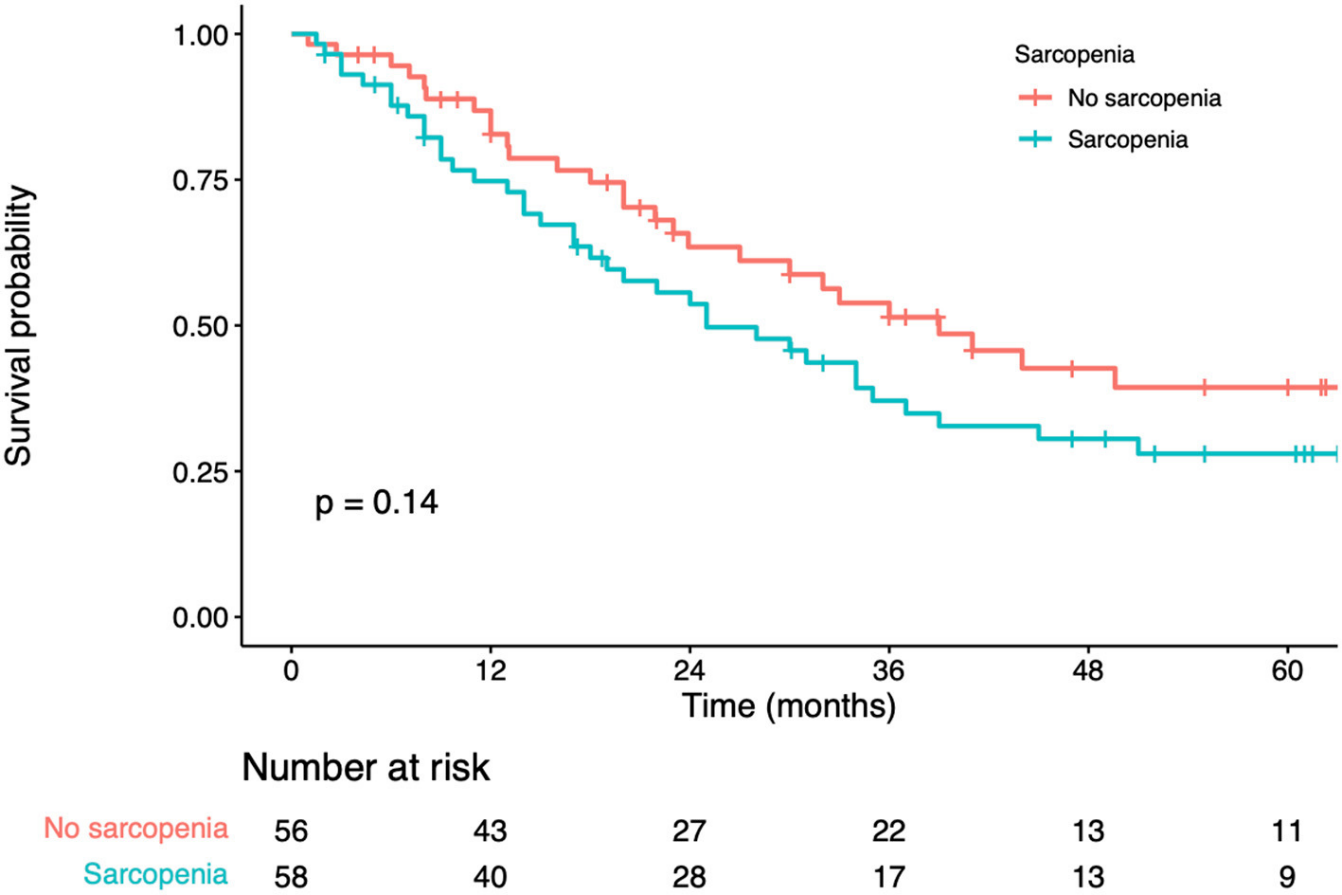
Kaplan-Meier curves for overall survival according to the preoperative sarcopenia status.

In univariate analysis, significant factors closely associated with OS included BMI (*p* = 0.010), serum albumin (*p* < 0.001), CEA levels (*p* < 0.001), number of liver metastases (*p* < 0.001), maximum diameter of the liver metastases (*p* < 0.001), adjuvant chemotherapy (*p* < 0.001), and sarcopenia status (*p* = 0.129) (Table 3). In multivariate analysis, serum albumin (hazard ratio [*HR*] = 1.63; 95% confidence interval [*CI*] = 1.12–2.48; *p* = 0.005), CEA levels (*HR* = 1.75; 95% *CI* = 1.26–2.29; *p* = 0.003), number of liver metastases (*HR* = 2.12; 95% *CI*=1.26–3.08; *p* < 0.01), maximum diameter of the liver metastases (*HR* = 1.54; 95% *CI*=1.09–2.85; *p* = 0.014) were the prognostic factors that independently predicted OS (Table 3). K-M curves displayed no significant difference in OS between sarcopenia and non-sarcopenia groups (log-rank test, *p*> 0.05) (Figure 2). We discovered that sarcopenia was not an independent prognostic factor for OS (*HR* = 1.62; 95% *CI*= 0.57–2.73; *p* = 0.330) (Table 3).

**Table 3:** Univariate and multivariate analysis of clinicopathologic variablesin relation to overall survivalin patients with GCLM after hepatectomy.

**Clinicopathological features**	**Univariate analysis**	***p*** **-value**	**Multivariate analysis**	***p*** **-value**
Age				
< 65.0 ≥ 65.0	Reference 1.36 (0.88, 2.69)	0.212		
Gender				
Male Female	Reference 0.83 (0.61, 2.75)	0.381		
BMI (kg/m^2^)				
≥ 18.5 < 18.5	Reference 2.32 (1.14, 3.81)	0.010	Reference 1.86 (0.85, 2.65)	0.204
Serum albumin (g/dL)				
≥ 3.5 < 3.5	Reference 1.97 (1.26, 3.11)	<0.001	Reference 1.63 (1.12, 2.48)	0.005
CEA (ng/mL)				
≥ 5.0 < 5.0	Reference 2.14 (1.38, 4.10)	<0.001	Reference 1.75 (1.26, 2.29)	0.003
ASA score				
1 2 3	Reference 1.37 (0.65, 3.87) 1.24 (0.70, 3.58)	0.632 0.309		
Tumor locationof GC				
Upper Middle/Lower	Reference 0.79 (0.54, 2.82)	0.241		
Lauren Classification				
Intestinal-type Diffused-type Mixed	Reference 2.21 (0.86, 3.84) 1.87 (0.71, 3.45)	0.304 0.445		
Tumor differentiation of GC				
G1 G2 G3	Reference 1.62 (0.76, 3.61) 1.83 (0.69, 3.20)	0.289 0.306		
Timing of liver metastases				
Metachronous Synchronous	Reference 1.54 (0.65, 2.79)	0.513		
Number of liver metastases				
≤ 1 >1	Reference 2.36 (1.35, 3.51)	<0.001	Reference 2.12 (1.26, 3.08)	<0.001
Maximum diameter of the liver metastasis				
<3 ≥ 3	Reference 1.89 (1.23, 3.29)	<0.001	Reference 1.54 (1.09, 2.85)	0.014
Type of hepatectomy				
Minor Major	Reference 1.14 (0.71, 1.71)	0.663		
Neoadjuvant chemotherapy				
No Yes	Reference 0.76 (0.45, 2.13)	0.217		
Adjuvant chemotherapy				
No Yes (Systemic chemotherapy/HAIC/ Systemic chemotherapy+HAIC)	Reference 0.68 (0.53, 0.97)	0.041	Reference 0.71 (0.55, 1.21)	0.189
Sarcopenia				
Without With	Reference 1.96 (0.92, 3.23)	0.129	Reference 1.62 (0.57, 2.73)	0.330

*GCLM, Gastric cancer liver metastasis; BMI, Body mass index; ASA, American society of anesthesiologists; GC, Gastric cancer; HAIC, Hepatic artery infusion chemotherapy*.

**Figure 3:**
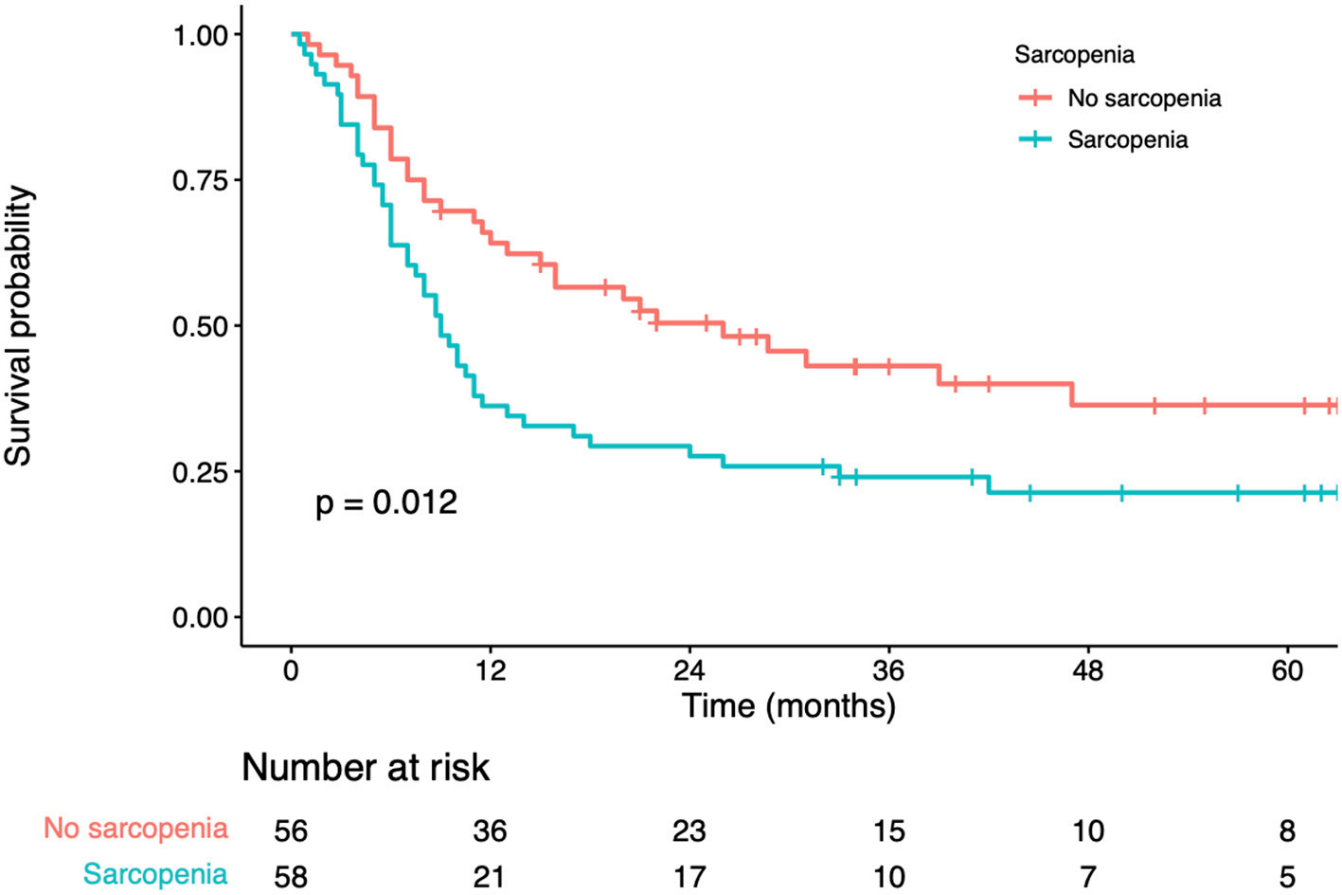
Kaplan-Meier curves for recurrence-free survival according to the preoperative sarcopenia status.

In univariate analysis, significant factors closely associated with RFS included serum albumin (*p* = 0.034), CEA levels (*p* = 0.011), number of liver metastases (*p* < 0.001), maximum diameter of the liver metastases (*p* = 0.002), sarcopenia status (*p* = 0.024) (Table 4). In multivariate analysis, number of liver metastases (*HR* = 1.88; 95% *CI*=1.12–2.72; *p* = 0.018), maximum diameter of the liver metastases (*HR* = 1.56; 95% *CI*=1.19–2.80; *p* = 0.011) were the prognostic factors that independently predicted RFS (Table 4). As revealed by K-M survival curves, sarcopenia predicted the dismal RFS (log-rank test, *p* < 0.001) (Figure 3). We discovered from multivariate analysis that sarcopenia independently predicted RFS (*HR*= 1.76; 95% *CI*= 1.18–2.35, *p* = 0.007) (Table 4).

**Table 4:** Univariate and multivariate analysis of clinicopathologic variables in relation to recurrence-free survival in patients with GCLM after hepatectomy.

**Clinicopathological features**	**Univariate analysis**	***p*** **-value**	**Multivariate analysis**	***p*** **-value**
Age				
< 65.0 ≥ 65.0	Reference 1.29 (0.81, 3.13)	0.309		
Gender				
Male Female	Reference 0.87 (0.58, 1.42)	0.620		
BMI (kg/m^2^)				
≥ 18.5 < 18.5	Reference 2.16 (0.62, 3.65)	0.238		
Serum albumin (g/dL)				
≥ 3.5 < 3.5	Reference 2.24 (1.06, 3.66)	0.034	Reference 1.60 (0.89, 2.51)	0.101
CEA (ng/mL)				
≥ 5.0 < 5.0	Reference 1.95 (1.20, 3.45)	0.011	Reference 1.54 (0.76, 2.03)	0.264
ASA score				
1 2 3	Reference 1.45(0.68, 2.65) 1.29 (0.54, 2.12)	0.710 0.522		
Tumor locationof GC				
Upper Middle/Lower	Reference 0.69 (0.51, 1.73)	0.719		
Lauren Classification				
Intestinal-type Diffused-type Mixed	Reference 1.91 (0.78, 2.16) 1.52 (0.56, 2.43)	0.451 0.304		
Tumor differentiation of GC				
G1 G2 G3	Reference 1.58 (0.72, 1.61) 1.74 (0.65, 2.28)	0.613 0.516		
Timing of liver metastases				
Metachronous Synchronous	Reference 1.67 (0.95, 3.52)	0.107		
Number of liver metastases				
≤1 >1	Reference 2.18 (1.63, 3.65)	<0.001	Reference 1.88 (1.12, 2.72)	0.018
Maximum diameter of the liver metastasis				
<3 ≥ 3	Reference 1.95 (1.27, 3.37)	0.002	Reference 1.56 (1.19, 2.80)	0.011
Type of hepatectomy				
Minor Major	Reference 0.87 (0.64, 1.42)	0.518		
Neoadjuvant chemotherapy				
No Yes	Reference 0.78 (0.52, 1.86)	0.326		
Adjuvant chemotherapy				
No Yes (Systemic chemotherapy/HAIC/ Systemic chemotherapy+HAIC)	Reference 0.75 (0.59, 1.26)	0.161		
Sarcopenia				
Without With	Reference 2.19 (1.07, 3.64)	0.024	Reference 1.76 (1.18, 2.35)	0.007

*GCLM, Gastric cancer liver metastasis; BMI, Body mass index; ASA, American society of anesthesiologists; GC, Gastric cancer; HAIC, Hepatic artery infusion chemotherapy*.

## Discussion

This study explored the prognostic significance of CT-determined sarcopenia among GCLM cases receiving liver resection. As a result, sarcopenia was identified as the prognostic index for predicting RFS for GCLM cases receiving liver resection independently. Based on our results, Sarcopenia was related to an advanced age (≥65.0 years), reduced BMI (<18.5 kg/m^2^) and number of liver metastases (>1).

Liver metastases can occur in approximately 5–14% of patients undergoing gastric cancer surgery (2). It is well known that the treatment of liver metastases is incredibly important for prognostic improvement in patients with GC. GC patients with liver metastases are traditionally receiving with palliative chemotherapy (23). Nevertheless, many studies report that surgical resection of liver metastases was related to a markedly improved survival in selected cases in the last couple of years. Survival rates of GCLM after hepatectomy from Far Eastern studies were higher than those from Western studies (2). Nonetheless, 5-year overall survival rates after hepatectomy range from 27.7 to 42.3 %, and recurrence rates range from 60 to 75 % (9, 24, 25). As a consequence, prediction and assessment of recurrence have become the extremely important considerations in patients with GCLM after liver resection. Sarcopenia, which has been confirmed as the losses of function and mass of skeletal muscle, predicts the dismal nutritional status. And it has been currently regarded as the tumor cachexia hallmark. Sarcopenia is clinically important among cancer cases, which arouses more and more interests from researchers in the last decade. Sarcopenia's prognostic significance is identified within different tumors. It was independently related to dismal prognosis of cases having GC (17). In addition, sarcopenia closely related to shorter progression-free survival following receiving immune checkpoint inhibitors in patients with malignancy, such as non-small cell lung cancer or renal cell carcinoma (26, 27). Sarcopenia was found to be an independent, unfavorable prognostic index for progression-free survival in advanced patients with GC receiving programmed death-1 inhibitor (28). Sarcopenia was confirmed as the independent prognostic index for OS and RFS in patients with hepato cellular carcinoma undergoing hepatectomy (29, 30). Previous studies indicate that colorectal cancer patients with sarcopenia have an inferior OS (31). Moreover, some of the studies indicated that sarcopenia was an independent predictor of long-term survival in colorectal liver metastases (CRLM) receiving liver resection (32–34). Additionally, van Dijk *et al*. found sarcopenia accompanied by elevated C-reactive protein was significantly related to a truncated OS in CRLM (35). It seems that these results contribute to the decisions concerning the timing of and the indications for liver resection. Our study showed that sarcopenia was not an independent prognostic factor for OS, although patients with sarcopenia had a trend toward dismal OS (log-rank test, *p* = 0.14). Although previous research indicated that sarcopenia can independently predicted OS among GC patients, this might not entirely be applicable in patients with GC, especially in patients with GCLM (22, 36). Our study confirmed the prognostic significance of sarcopenia in GCLM cases receiving liver resection, that sarcopenia independently predicted RFS. Sarcopenia contributes to accurately predicting the prognosis and assisting decision-making among GCLM cases. Meanwhile, it can be utilized as one part of prognosis stratification before surgery, and sarcopenia predicts an increased tumor recurrence risk and the necessity for customized treatment.

The mechanism by which sarcopenia increases the risk of tumor recurrence is still obscure. The following reasons can be assumed. First, sarcopenia may reflect increased metabolic activity of more aggressive tumor biology, which leads to more severe systemic inflammation and subsequent muscle wasting (37). Second, previous studies reported that myokines secreted by muscle cells can inhibit the growth of cancer cells. Therefore, we speculate that a reduction in muscle mass may lead to an impaired myokine response and increases the risk of tumor recurrence (38).

There are some limitations in the present work. First, the AWGS (2019 edition) suggests using the presence of loss of muscle quality plus low muscle function (strength or performance) to determine sarcopenia. Because our study design was retrospective in nature, it was not possible to collect information on muscle function (muscle strength or physical performance). Therefore, we preoccupied with muscle quality assessment to identify patients with sarcopenia. CT is used as a means to accurately assess muscle mass. There are several advantages to the use of CT. CT is widely used as a routine examination and staging method for patients with gastric cancer and can accurately quantify muscle mass. The definition of sarcopenia put forward by Zhuang *et al*. was utilized in the present work, which defined sarcopenia criteria for Chinese population (22). The L3-SMI thresholds for diagnosing CT-based sarcopenia were 34.9 cm^2^/m^2^ and 40.8 cm^2^/m^2^ for women and men, separately. This study showed limited generalizability to western populations, since our adopted L3 SMI thresholds showed high specificity to geographic location. Secondly, the effect of preoperative sarcopenia on predicting the prognosis of GCLM cases receiving hepatectomy was evaluated, but selection bias still existed due to the retrospective nature. And we only recruited cases at a single center in China, showing ethic homogeneity. For overcoming the above limitations, more large-scale multicenter prospective studies should be conducted.

## Conclusions

In conclusion, we suggest that CT-determined sarcopenia is a meaningful predictor of recurrence after hepatectomy in patients with GCLM, and it should be regarded as one of the assessment criteria of hepatectomy.

## Data Availability Statement

The original contributions presented in the study are included in the article/Supplementary Material, further inquiries can be directed to the corresponding author.

## Ethics Statement

The studies involving human participants were reviewed and approved by the Institutional Review Board of National Cancer Center/Cancer Hospital, Chinese Academy of Medical Sciences and Peking Union Medical College. Written informed consent for participation was not required for this study in accordance with the national legislation and the institutional requirements.

## Author Contributions

JX conceived the study and wrote the manuscript. YW, WK, and HH searched the database, reviewed the studies and collected the data. XS and JX performed the statistical analyses. YL, PJ, and WL performed revision of the manuscript. YT arranged for and provided the funding for this work. All authors reviewed the manuscript and participated in its revision. YT had full access to all of the data in the study and takes responsibility for the integrity of the data and the accuracy of the data analysis. All authors of this manuscript have read and approved the final submitted version and are aware that they are listed as an author on this paper.

## Funding

This work was supported by grants from the National Natural Science Foundation of China (81772642).

## Conflict of Interest

The authors declare that the research was conducted in the absence of any commercial or financial relationships that could be construed as a potential conflict of interest.

## Publisher's Note

All claims expressed in this article are solely those of the authors and do not necessarily represent those of their affiliated organizations, or those of the publisher, the editors and the reviewers. Any product that may be evaluated in this article, or claim that may be made by its manufacturer, is not guaranteed or endorsed by the publisher.

## Abbreviations

GC: Gastric cancer
GCLM: Gastric cancer liver metastasis
BMI: Body mass index
ASA: American society of anesthesiologists
GC: Gastric cancer
HAIC: Hepatic artery infusion chemotherapy
OS: Overall survival
RFS: Recurrence-Free survival
AWGS: Asian working group for sarcopenia
SMI: Skeletal muscle index
CT: Computed tomography
HU: Hounsfield units
L3: Third lumbar vertebra
CSA: Cross-Section area
HR: Hazard ratio
95% *CI*: 95% Confidence interval
CRLM: Colorectal liver metastases.
